# Paeoniflorin Ameliorates Cecal Ligation and Puncture-Induced Acute Lung Injury in Mice by Modulating Oxidative Stress, Apoptosis, and Inflammation: Shedding Light on the Role of the JAK2/STAT3 Pathway

**DOI:** 10.3390/ph19050666

**Published:** 2026-04-24

**Authors:** Nourhan Hisham Shady, Reham H. Mohyeldin, Nehad M. Reda Abdel Maqsoud, Peter A. Sidhom, Mahmoud A. A. Ibrahim, Ahmed M. Shawky, Mohamed Hisham, Gerhard Bringmann, Usama Ramadan Abdelmohsen, Dalia H. Abu-Baih

**Affiliations:** 1 Department of Pharmacognosy, Faculty of Pharmacy, Deraya University, Universities Zone, New Minia 61111, Egypt; 2 Center for Entrepreneurship and Research in Sustainability, Deraya University, Universities Zone, New Minia 61111, Egypt; 3 Department of Pharmacology & Toxicology, Faculty of Pharmacy, Deraya University, New Minia 61111, Egypt; reham.hassan@deraya.edu.eg; 4 Department of Pathology, Deraya University, New Minia 61111, Egypt; 5 Department of Pathology, Faculty of Medicine, Minia University, Minia 61519, Egypt; 6 Department of Pharmaceutical Chemistry, Faculty of Pharmacy, Tanta University, Tanta 31527, Egypt; 7 School of Health Sciences, University of KwaZulu-Natal, Westville Campus, Durban 4000, South Africa; 8 Department of Supportive Requirements, University of Technology and Applied Sciences, Nizwa 611, Oman; 9 Department of Chemistry, Faculty of Science, Umm Al-Qura University, Makkah 21955, Saudi Arabia; amesmail@uqu.edu.sa; 10 Department of Pharmaceutical Chemistry, Faculty of Pharmacy, Deraya University, New Minia 61111, Egypt; 11 Institute of Organic Chemistry, University of Würzburg, Am Hubland, 97074 Würzburg, Germany; gerhard.bringmann@uni-wuerzburg.de; 12 Department of Pharmacognosy, Faculty of Pharmacy, Minia University, Minia 61519, Egypt; 13 Department of Biochemistry and Molecular Biology, Faculty of Pharmacy, Deraya University, New Minia 61111, Egypt

**Keywords:** CLP, sepsis, JAK2/STAT3, paeoniflorin, network pharmacology

## Abstract

**Background:** Acute lung injury (ALI) is a major complication of sepsis, driven by oxidative stress, inflammation, and apoptosis. Paeoniflorin, a monoterpenoid glycoside, has demonstrated notable antioxidant and anti-inflammatory properties, suggesting potential therapeutic value in ALI. **Methods:** Sepsis-induced ALI was established in mice using the cecal ligation and puncture (CLP) model. The protective effects of paeoniflorin were evaluated by measuring oxidative stress markers (SOD, GSH, and MDA) and pro-inflammatory cytokines (TNF-α, IL-6, and IL-1β) using biochemical assays and RT-PCR. Histopathological examination and apoptosis assessment (Bax and Bcl-2 expression) were performed. Western blot analysis was conducted to investigate the involvement of the JAK2/STAT3 signaling pathway. Network pharmacology analysis was used to identify potential molecular targets, and molecular docking was performed to explore binding interactions. **Results:** CLP-induced ALI resulted in increased oxidative stress and inflammatory responses, as evidenced by elevated MDA and cytokine levels, along with reduced SOD and GSH levels. Paeoniflorin treatment significantly ameliorated these alterations. Histological damage and apoptosis were markedly reduced, accompanied by the downregulation of Bax and upregulation of Bcl-2. Additionally, paeoniflorin inhibited activation of the JAK2/STAT3 pathway. Network pharmacology identified key ALI-related targets, including IL6, TNF, IL1B, HIF1A, STAT3, NFKB1, CCL2, CYBB, CXCL8, and NOX4. Molecular docking revealed strong binding affinity of paeoniflorin toward HIF-1 and JUN, and moderate interactions with IL-1β, TNF-α, and Bax. **Conclusions:** Paeoniflorin exerts protective effects against sepsis-induced ALI by attenuating oxidative stress, inflammation, and apoptosis, partly through inhibition of the JAK2/STAT3 signaling pathway. These findings highlight its potential as a promising therapeutic candidate for ALI management.

## 1. Introduction

Sepsis is described as a critical organ dysfunction resulting from an abnormal host response to infection [1,2]. Annually, over 18 million cases of sepsis are reported worldwide, indicating an incidence rate ranging from 1.5% to 8.0% per year. Sepsis is a highly lethal condition, accounting for an estimated 14,000 fatalities daily on a global scale as a result of complications [3]. Sepsis is characterized by elevated mortality rates, increased healthcare expenditures, and greater rates of readmission following discharge, in comparison to non-septic diseases [4]. Consequently, exploring the pathophysiological causes of sepsis and formulating novel therapeutic methods are urgent scientific research priorities [5]. Sepsis causes substantial morbidity and mortality, primarily attributable to acute respiratory distress syndrome (ARDS) and acute lung injury (ALI) [6] resulting in multiple organ dysfunction. Sepsis-related multiple organ dysfunctions, significant hypotension, and coagulation abnormalities are the main risk factors for the onset of ALI [7]. Severe hypoxemia, decreased lung compliance, alveolar inundation with protein-rich edema, and elevated vascular permeability accompanied by diffuse bilateral pulmonary infiltrates are additional hallmarks of ALI in septic condition [8,9].

The pathophysiology of ALI is multifaceted, with oxidative stress, inflammation, and apoptosis. Oxidative stress plays a role in inflammation, which can lead to tissue damage and hurt the progression of sepsis cases [10]. The equilibrium between oxidants and antioxidant scavengers is an important issue and has to be maintained. However, this delicate balance is disrupted when the concentration of oxidants in the cells rises, leading to dysfunction in multiple organs [11]. Abnormal oxidant production plays a crucial role in septicemia. In the latest studies of animal models, a correlation between sepsis and changes in oxidative stress markers has been discovered. Malondialdehyde (MDA) levels, as biomarkers for sepsis, are elevated, while the activities of superoxide dismutase (SOD) and glutathione (GSH) enzymes are decreased [12].

Apoptosis is a cellular process designed to eliminate cells that have been rendered pathologically compromised or pose a risk to cellular equilibrium [13]. Furthermore, endothelial and epithelial barrier dysfunction is induced by excessive alveolar cell apoptosis in response to a variety of environmental stimuli, which in turn promotes ALI [14].

Primary neutrophils and inflammatory cells accumulate and activate in the lung [15]. Pro-inflammatory cytokines, including IL-1β, TNF-α, and IL-6, are synthesized by inflammatory cells and are commonly found in the lung, hence intensifying the ALI inflammatory response and aggravating lung tissue damage.

In septic ALI/ARDS, pulmonary microvascular leakage is a crucial indicator of barrier dysfunction. The pathogenesis of endothelial barrier dysfunction encompasses numerous pathways that are associated with oxidative stress and inflammatory response [16]. It is hypothesized that lipopolysaccharide derived from Gram-negative bacteria acts as an endotoxin via a cascade of innate immune responses triggered by its binding to a receptor on the cell membrane. Proximous and potent induction of ROS and inflammatory factors by LPS exposure results in endothelial barrier disruption and increase microvascular permeability. Signaling molecules that modulate oxidative stress and the inflammatory response are crucial for the prevention and management of sepsis-induced ALI [17]. In spite of recent developments in intensive care and antibiotic therapy, the prognosis for ALI continues to be dismal. Thus, immediate action is required to prevent sepsis and septic complications [18]. Despite accumulating evidence supporting the anti-inflammatory and antioxidant properties of paeoniflorin, its mechanistic role in sepsis-induced acute lung injury (ALI), particularly within clinically relevant models such as cecal ligation and puncture (CLP), remains insufficiently characterized. Unlike endotoxin- or virus-induced models, the CLP model more closely recapitulates the complex and systemic nature of human sepsis, including the interplay between inflammatory, oxidative, and apoptotic pathways. Moreover, the involvement of key signaling cascades, particularly the JAK2/STAT3 pathway, in mediating the protective effects of paeoniflorin has not been fully elucidated. In addition, most previous studies have focused on isolated biological effects without integrating computational and experimental approaches. Therefore, the present study aims to provide a comprehensive and integrative investigation by combining network pharmacology, molecular docking, and in vivo validation to delineate the multi-target mechanisms underlying paeoniflorin-mediated protection against CLP-induced lung injury. This approach not only enhances mechanistic understanding, but also supports the potential translational relevance of paeoniflorin as a multi-pathway therapeutic agent in sepsis-associated ALI.

**Figure 1:**
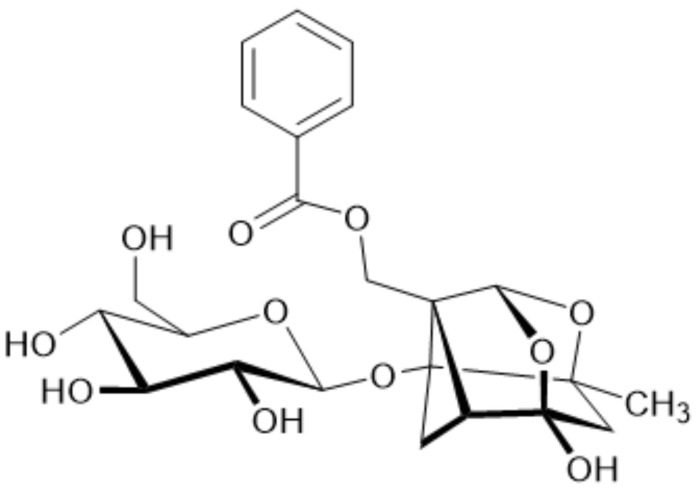
Structure of paeoniflorin.

Paeoniflorin, a hydrophilic monoterpene glycoside shown in Figure 1, stands as the principal bioactive constituent within Peony plants. The scientific literature underscores its multifaceted pharmacological prowess, showcasing anti-inflammatory, antioxidant, and immunomodulatory properties [19]. While research has demonstrated the efficacy of paeoniflorin in alleviating ALI induced by the influenza virus in an animal model [20], scant attention has been given to its potential impact on ALI triggered by cecal ligation and puncture (CLP). To establish a robust theoretical foundation for employing paeoniflorin as a prophylactic measure against sepsis-induced acute lung injury (ALI), this study endeavored to scrutinize the impact of paeoniflorin on survival rates within a murine sepsis model. Additionally, it sought to explore the role of the drug and potential mechanisms in acute lung damage in septic mice.

Given the complex, multifactorial pathogenesis of sepsis-induced ALI, single-target approaches are often insufficient. Network pharmacology provides a systems-level strategy to identify multi-target interactions and signaling networks associated with bioactive compounds. Molecular docking further complements this approach by validating the binding affinity between compounds and predicted targets at the structural level. In this study, targets selected for molecular docking were derived from hub genes identified through network analysis, particularly those involved in inflammatory and JAK2/STAT3-related pathways. Therefore, integrating these computational approaches with in vivo validation enables a more comprehensive understanding of the pharmacological mechanisms of paeoniflorin and facilitates the identification of key pathways for experimental investigation.

## 2. Results

### 2.1. Computational Study

#### 2.1.1. Network Pharmacology-Based Analysis

To elucidate the mechanisms and pathways through which paeoniflorin exerts protective effects on acute lung injury, a network pharmacology analysis was employed. This method maps interactions within biological networks, thereby uncovering how drugs influence various pathways to produce therapeutic outcomes.

#### 2.1.2. Screening of Paeoniflorin Related Targets Genes

Utilizing the TCMSP, Batman-TCM, and SwissTargetPrediction databases, a total of 159 targets related with paeoniflorin were identified. These databases were selected due to their comprehensive integration of pharmacokinetic data, chemical similarity prediction, and known drug–target interactions. For SwissTargetPrediction, targets with a probability score > 0.5 were selected to ensure higher prediction confidence, while lower-confidence predictions were excluded. These genes were subsequently standardized to official gene names using the UniProt database. Redundant entries were removed to ensure data consistency.

#### 2.1.3. Screening of Acute Lung Injury Related Genes

**Figure 2:**
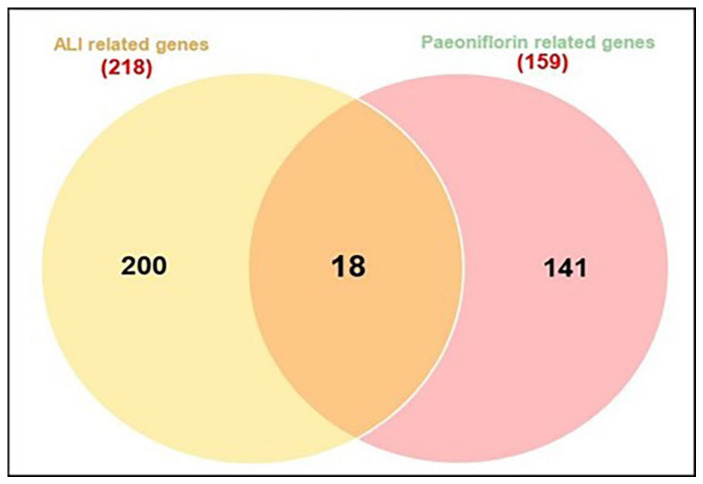
Venn diagram for the integrated analysis of the related targets of paeoniflorin and ALI targets.

A comprehensive search of the NCBI, GeneCards, and DisGeNET databases, using the term “acute lung injury” and restricting results to “*Homo sapiens*”, identified 218 pharmacological targets associated with acute lung injury. To improve specificity, genes with higher relevance scores (≥0.7) were prioritized, and duplicate entries were removed. An interactive Venn diagram was then used to compare these targets with those modulated by paeoniflorin, revealing a total of 18 intersecting genes after the removal of duplicates (Figure 2).

#### 2.1.4. Protein–Protein Interaction (PPI) Network Construction

**Figure 3:**
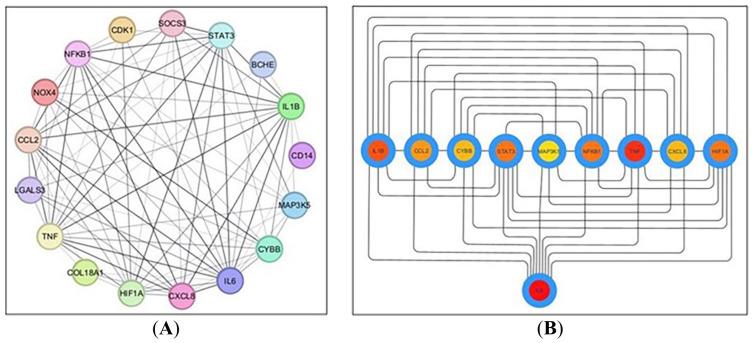
(**A**) Network nodes represent 17 protein targets, and the edges represent protein–protein interactions; (**B**) network nodes represent the top ten hub genes: the darker the color, the higher the score and the stronger the connection.

The 18 shared targets identified from the Venn diagram were analyzed for their interrelationships using the STRING database (version 12.0) with a minimum required interaction score of 0.400 (medium confidence) and the organism set to *Homo sapiens*. A PPI network was then constructed in Cytoscape v 3.10.2, resulting in 17 connected nodes (after excluding one unconnected node) and 84 edges, with an average node connectivity of 9.882, as depicted in Figure 3A. Subsequently, the CytoHubba plugin was used to identify and extract the ten most prominent genes based on degree centrality, which ranks nodes according to the number of their direct interactions within the network, as shown in Figure 3B. These genes include IL6, TNF, IL1B, HIF1A, STAT3, NFKB1, CCL2, CYBB, CXCL8, and NOX4. Collectively, the ten hub genes identified by CytoHubba degree centrality analysis represent functionally interconnected components of the major pathological axes of ALI: (i) the pro-inflammatory cytokine cascade (IL6, TNF, IL1B, CXCL8, CCL2), (ii) the transcriptional regulatory core (NFKB1, STAT3), (iii) the hypoxia-sensing response (HIF1A), and (iv) the NADPH oxidase-mediated oxidative stress machinery (CYBB, NOX4). Their high network connectivity reflects the extensive molecular crosstalk among these axes in sepsis-induced ALI and provides a mechanistic rationale for the multi-target therapeutic approach explored in this study.

The topological parameters for each of these proteins, including node degree, betweenness, and closeness, are summarized in Table 1.

#### 2.1.5. GO and KEGG Enrichment Analyses

**Figure 4:**
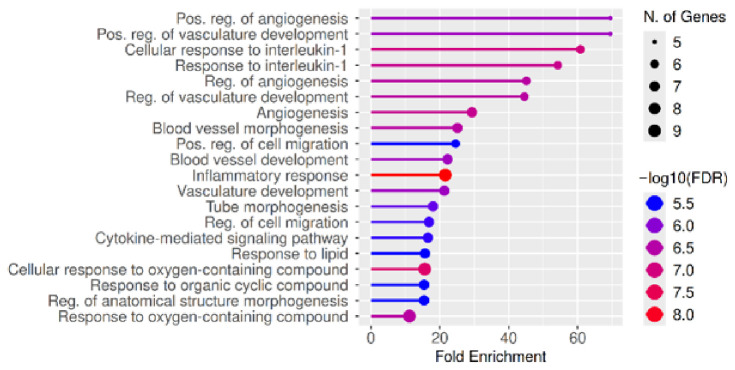
Gene Ontology (GO) enrichment analysis of the top ten hub genes associated with acute lung injury. The enriched biological processes are shown on the y-axis, while the x-axis represents the fold enrichment. The size of each dot corresponds to the number of genes involved in each term, and the color gradient indicates the significance level (−log10 FDR).

GO and KEGG enrichment analyses were performed to further elucidate the functional roles of the identified hub genes in acute lung injury. GO analysis revealed that these genes are significantly involved in biological processes related to inflammation, immune response, oxidative stress, and vascular development, including cytokine-mediated signaling, response to interleukin-1, cell migration, and angiogenesis. These processes are closely associated with the key pathological features of ALI, such as endothelial dysfunction, immune cell infiltration, and tissue remodeling (Figure 4).

**Figure 5:**
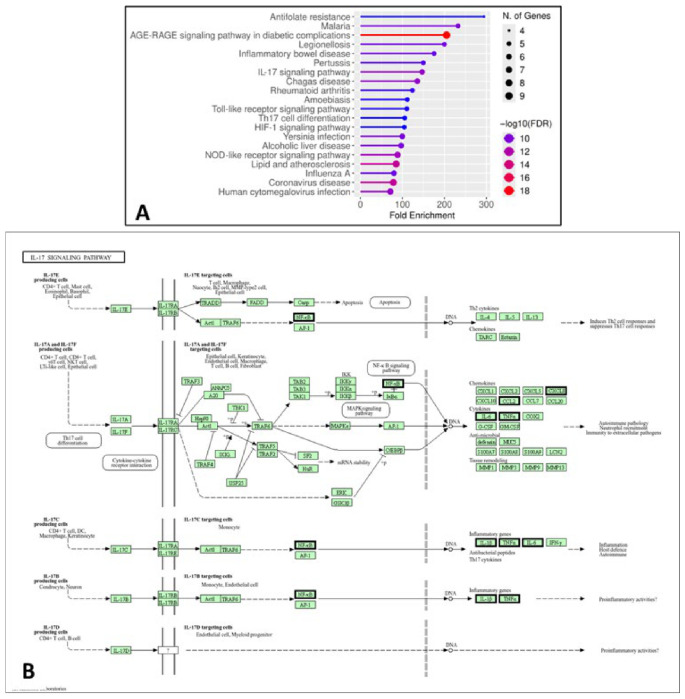
KEGG pathway enrichment and IL-17 signaling analysis of hub genes. (**A**) KEGG enrichment showing significant pathways related to inflammation and immune response, with dot size indicating gene count and color representing significance (−log10 FDR). (**B**) IL-17 signaling pathway map highlighting hub genes (bold edges) involved in inflammatory regulation relevant to acute lung injury.

Consistent with these findings, KEGG pathway analysis demonstrated significant enrichment in pathways associated with inflammatory and immune signaling, including the IL-17 signaling pathway, Toll-like receptor signaling pathway, and NOD-like receptor signaling pathway. These pathways regulate the activation of key transcription factors such as NF-κB and AP-1, leading to the increased expression of pro-inflammatory cytokines (e.g., IL-6, TNF-α, and IL-1β) and chemokines that promote neutrophil recruitment and amplify inflammatory responses. In addition, enrichment of the HIF-1 signaling pathway highlights the role of hypoxia in ALI, while pathways such as AGE-RAGE signaling and lipid and atherosclerosis further reflect the contribution of oxidative stress and endothelial dysfunction (Figure 5). Collectively, these results indicate that the identified hub genes are involved in interconnected biological processes and signaling pathways that drive inflammation, oxidative stress, hypoxia, and immune dysregulation in ALI. These findings are consistent with our experimental observations and support the hypothesis that paeoniflorin exerts its protective effects through multi-target and multi-pathway regulation.

#### 2.1.6. Molecular Docking

To investigate the interactions of paeoniflorin with target proteins, we utilized molecular docking, hypothesizing potential protective effects against acute lung injury. For this study, we selected the top five hub genes (IL6, TNF, IL1B, HIF1A, and STAT3) identified from a pharmacological network analysis, in addition to JUN and Bax, for the docking investigation. To ensure the validity of our docking approach, we re-docked co-crystallized ligands and confirmed that the RMSD between the crystal and docked poses was less than 2 Å. We then docked paeoniflorin into these target proteins, with the resulting docking scores presented in Table 2.

**Figure 6:**
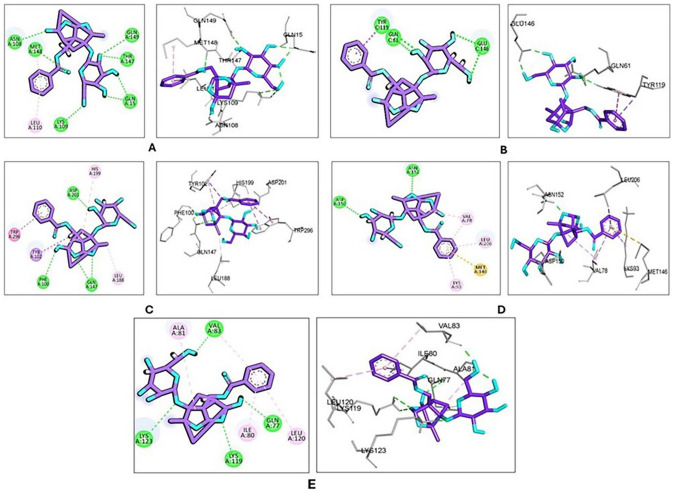
2D and 3D molecular docking of paeoniflorin with the highest obtained binding poses with (**A**) IL-1β; (**B**) TNF-α; (**C**) HIF-1; (**D**) JUN; and (**E**) Bax. Paeoniflorin is shown as purple sticks. Amino acid residues are represented in gray sticks. Green dashed lines indicate hydrogen bonds, while pink dashed lines represent π-interactions.

The docking scores of the co-crystallized ligands were compared to those of paeoniflorin, noting that lower scores indicate higher binding affinities. Paeoniflorin exhibited moderate docking scores across several targets associated with inflammation and cellular regulation. Notably, it showed relatively better binding affinity toward HIF-1 (docking score = −5.25) and JUN (docking score = −5.80) compared to other targets; however, these affinities were lower than those of the co-crystallized ligands. These findings suggest possible interactions that may contribute to its biological effects, although they should be interpreted with caution. Additionally, paeoniflorin was found to have moderate affinities for IL-1β (docking score = −4.03), TNF-α (docking score = −4.36), and Bax (docking score = −4.38), supporting their potential involvement in the observed anti-inflammatory and anti-apoptotic pathways, as illustrated in Figure 6A–E; the binding interactions are summarized in Table 3.

### 2.2. Survival Rates in Septic Mice

**Figure 7:**
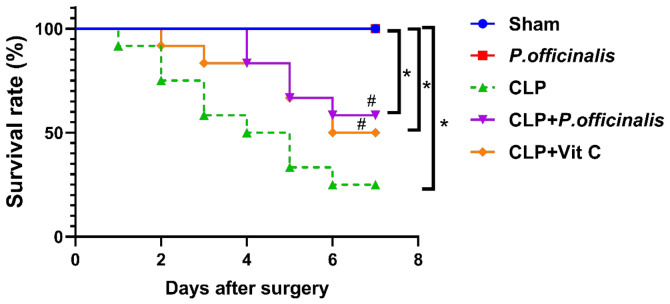
Survival analysis of experimental groups following CLP. Kaplan–Meier survival curves showing the survival probability of mice over a 7-d period after CLP induction. Data represent *n* = 10 animals per group. Survival differences were analyzed using the log-rank (Mantel–Cox) test. Significance was at * *p*  <  0.05 vs. sham group, ^#^ *p*  <  0.05 vs. CLP group.

To investigate the efficacy of paeoniflorin in an animal model of sepsis, mice were monitored for a duration of 7 d following CLP. Kaplan–Meier survival analysis demonstrated significant differences among the experimental groups over the 7-d observation period. Consistent with these findings, the CLP group exhibited a markedly reduced survival rate (25%) compared to the sham group (100%). Pairwise comparisons using the log-rank (Mantel–Cox) test confirmed a significant reduction in survival in the CLP group relative to the sham group (*p* = 0.002). Treatment with paeoniflorin improved survival, with a survival rate of 58%, and this improvement was statistically significant compared to the CLP group (*p* = 0.0474), indicating a protective effect against CLP-induced mortality. However, survival in the CLP + paeoniflorin group remained significantly lower than that of the sham group (*p* = 0.0137), suggesting a partial but incomplete restoration of survival. Notably, no statistically significant difference was observed between the paeoniflorin-treated and vitamin C-treated groups (*p* = 0.696), indicating comparable efficacy between the two interventions as shown in Figure 7.

### 2.3. Histopathological Examination

**Figure 8:**
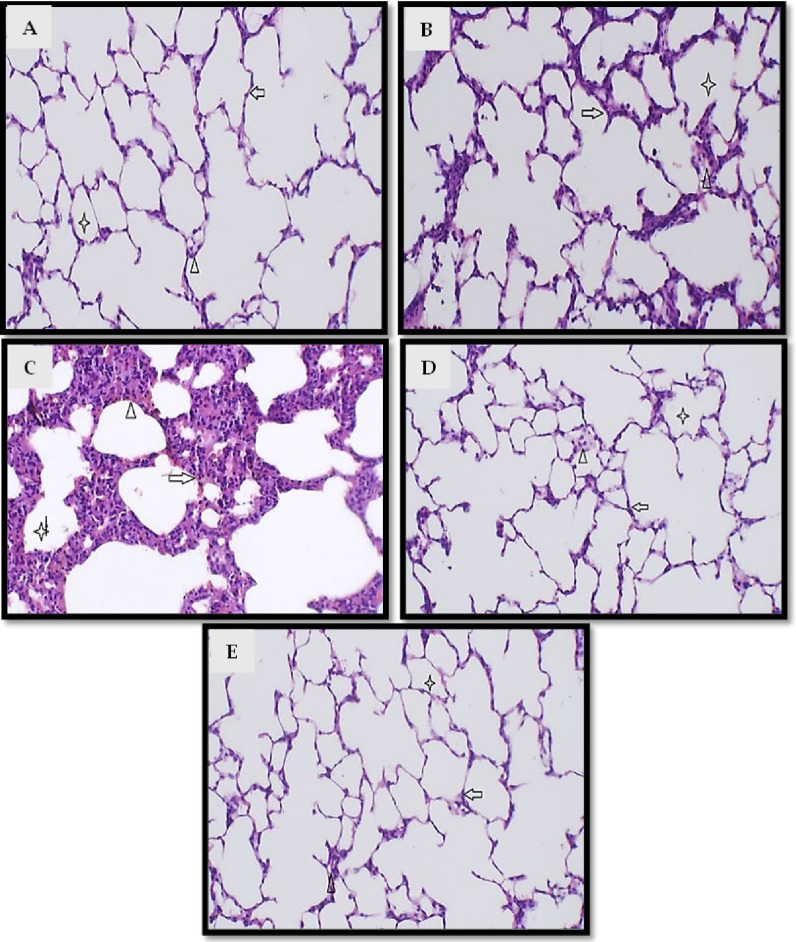
Histopathological examination of lung tissues (H&E staining). Representative photomicrographs of lung tissue sections from (**A**) sham group, (**B**) paeoniflorin group, (**C**) CLP group, (**D**) CLP + paeoniflorin group, and (**E**) CLP + vitamin C group; (**A**) section in lung tissue showed normal alveoli with simple cuboidal lining (arrow), normal interalveolar space with non-congested capillaries (arrowhead), and normal intra-alveolar space without slough or congestion (asterisk); (**B**) section in lung tissue showed normal alveoli with simple cuboidal lining (arrow), normal interalveolar space with non-congested capillaries (arrowhead), and normal intra-alveolar space without slough or congestion (asterisk); (**C**) section in lung tissue showed marked thickened alveolar membrane with dense inflammatory cellular infiltrates formed mainly of neutrophils (arrow), congested and inflamed interalveolar spaces (arrowhead), normal intra-alveolar space without slough or congestion with dilatation (asterisk); (**D**,**E**) sections in lung tissue showed normal alveoli with simple cuboidal lining (arrow), normal interalveolar space with non-congested capillaries (arrowhead), and normal intra-alveolar space without slough or congestion (asterisk).

Figure 8 presents the histological results of the lung tissues investigated. Tissues from the control and paeoniflorin groups exhibited normal histological lung structures (Figure 8A and Figure 8B, respectively). Inversely, lung tissues of the CLP group showed marked thickened alveolar membranes with dense inflammatory cellular infiltrates formed mainly of neutrophils with congested and inflamed interalveolar spaces. Normal intra-alveolar space without slough or congestion with dilatation was also observed (Figure 8C). The paeoniflorin + CLP and vitamin C + CLP groups showed that sections in lung tissue showed normal alveoli with simple cuboidal lining, normal interalveolar space with non-congested capillaries, and normal intra-alveolar space without slough or congestion (Figure 8D,E).

### 2.4. Evaluation of Total Cell Count, Neutrophil Infiltration, LDH Activity, and Total Protein Content in BALF

**Figure 9:**
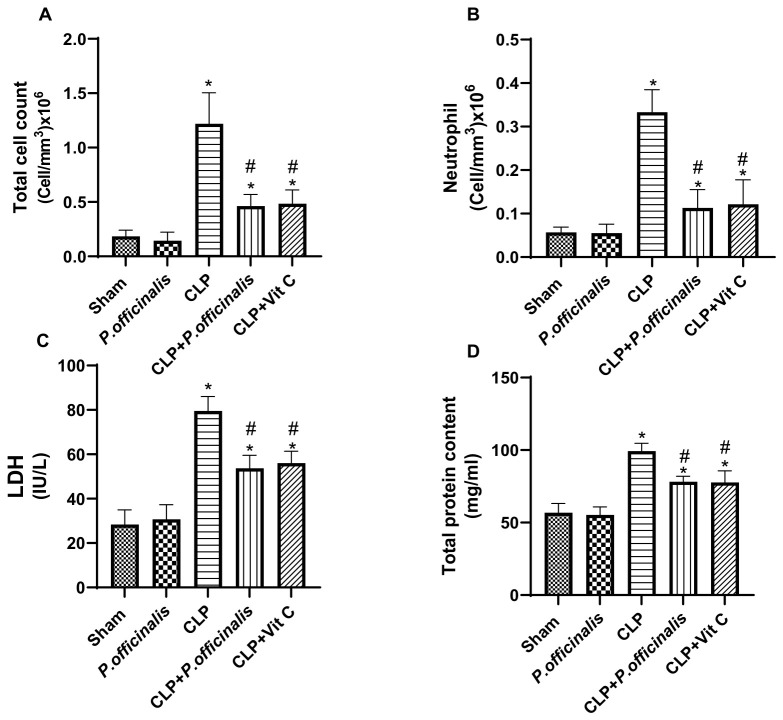
Effect of paeoniflorin on BALF parameters. (**A**) Total cell count, (**B**) neutrophil count, (**C**) lactate dehydrogenase (LDH) activity, and (**D**) total protein content in bronchoalveolar lavage fluid (BALF). Data are expressed as the mean ± SD (*n* = 6). Statistical analysis was performed using one-way ANOVA followed by Tukey’s post hoc test. * *p* < 0.05 vs. sham group, # *p* < 0.05 vs. CLP group.

CLP induction resulted in a pronounced inflammatory and injury response in bronchoalveolar lavage fluid (BALF), as evidenced by a marked increase in total cell count (Figure 9A), which reached an amount approximately 6.7-fold higher than the sham group, reflecting substantial inflammatory cell recruitment. This was accompanied by a significant elevation in neutrophil infiltration (Figure 9B; ~5.9-fold vs. sham), indicating a robust neutrophil-driven inflammatory response. In addition, LDH activity (Figure 9C) was significantly increased (~2.8-fold vs. sham), reflecting enhanced cellular injury, along with a concomitant rise in total protein content (Figure 9D; ~1.7-fold vs. sham), suggesting increased alveolar-capillary barrier permeability.

Treatment with paeoniflorin significantly attenuated these alterations, reducing the total cell count (Figure 9A) to approximately 0.38-fold of the CLP group (≈62% reduction) and neutrophil infiltration (Figure 9B) to approximately 0.34-fold of CLP (≈66% reduction), indicating marked suppression of inflammatory cell recruitment. Moreover, LDH activity (Figure 9C) and total protein levels (Figure 9D) were reduced to approximately 0.67-fold and 0.79-fold of CLP, respectively, demonstrating attenuation of tissue injury and restoration of alveolar-capillary barrier integrity. A comparable pattern of improvement was observed in the vitamin C-treated group, further supporting the protective effects of both interventions on lung inflammation and injury.

### 2.5. Effect of Paeoniflorin on Pulmonary Edema (W/D Ratio)

**Figure 10:**
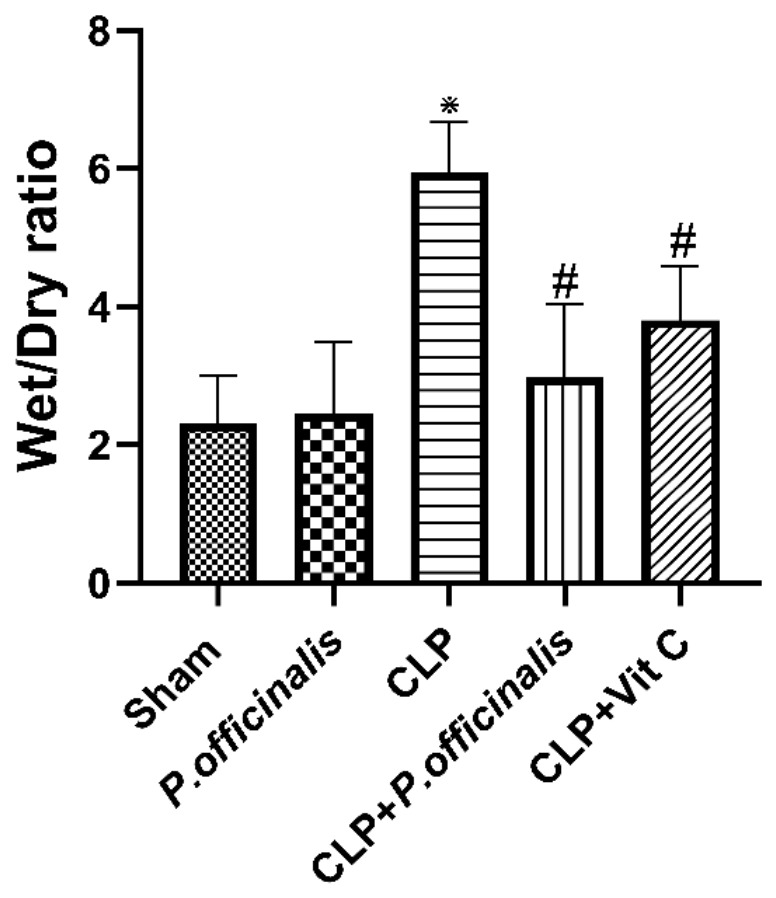
Effect of paeoniflorin on lung edema. Lung wet-to-dry (W/D) weight ratio as an indicator of pulmonary edema. Data are expressed as the mean ± SD (*n* = 6). Statistical analysis was performed using one-way ANOVA followed by Tukey’s post hoc test. * *p* < 0.05 vs. sham group, # *p* < 0.05 vs. CLP group.

As illustrated in Figure 10, the CLP group exhibited a marked increase in lung wet-to-dry (W/D) ratio, reaching approximately 2.6-fold higher than the sham group, indicating severe pulmonary edema. Treatment with paeoniflorin significantly attenuated this increase, reducing the W/D ratio to approximately 0.5-fold of the CLP group (≈50% reduction), approaching near-baseline levels. A comparable reduction (~0.64-fold of CLP) was observed in the vitamin C-treated group, confirming a substantial protective effect against fluid accumulation in lung tissue.

### 2.6. Oxidative Stress Assessment

**Figure 11:**
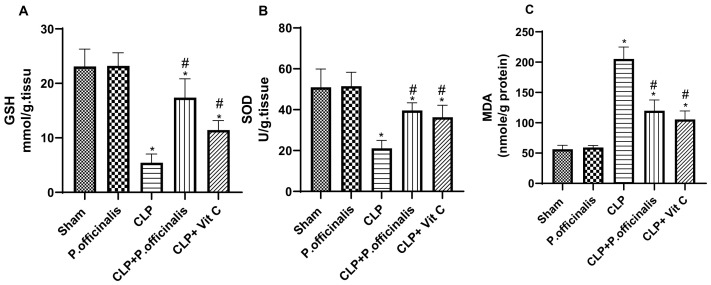
Effect of paeoniflorin on oxidative stress markers in lung tissue. (**A**) Reduced glutathione (GSH), (**B**) superoxide dismutase (SOD), and (**C**) malondialdehyde (MDA) levels in lung tissue homogenates. Data are expressed as the mean ± SD (*n* = 6). Statistical analysis was performed using one-way ANOVA followed by Tukey’s post hoc test. * *p* < 0.05 vs. sham group, # *p* < 0.05 vs. CLP group.

Figure 11A–C demonstrates that the mice in the CLP group had the highest MDA levels, accompanied by a substantial reduction in SOD and GSH compared to the sham group. Mice treated with paeoniflorin exhibited a substantial enhancement in the activity of the aforementioned antioxidants and a considerable reduction in MDA levels relative to the control group.

### 2.7. mRNA Levels of IL-6, TNF-α, and IL-1β

**Figure 12:**
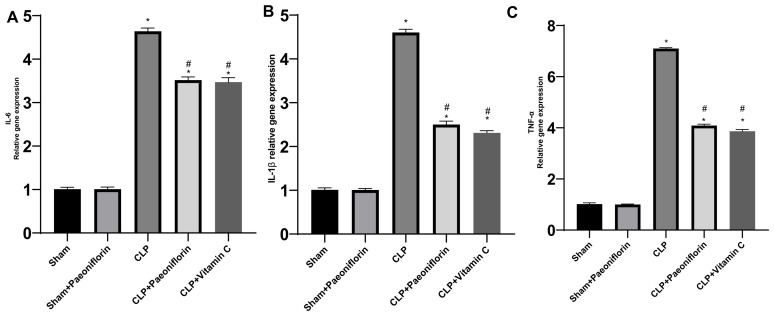
Effect of paeoniflorin on pro-inflammatory cytokine gene expression. Relative mRNA expression levels of (**A**) IL-6, (**B**) IL-1β, and (**C**) TNF-α in lung tissues, measured by qRT-PCR and normalized to GAPDH. Data are expressed as fold change relative to the sham group (mean ± SD, *n* = 6). * *p* < 0.05 vs. sham group, # *p* < 0.05 vs. CLP group.

To assess the level of inflammatory cytokines in the lung tissue of mice, qRT-PCR was employed. Figure 12 illustrates a substantial rise in the expression of IL-6, TNF-α, and IL-1β in the CLP group (4.64-fold, 7.1-fold, and 4.6-fold, respectively) compared to the sham group (*p*  <  0.05). The CLP  +  paeoniflorin group exhibited substantial inhibition of IL-6, TNF-α, and IL-1β gene expression, with reductions of 3.51-fold, 4.08-fold, and 2.5-fold (*p*  <  0.05), relative to the CLP mice. The CLP + vitamin C group demonstrated results analogous to the CLP + paeoniflorin group, with increases of 3.47-fold, 3.86-fold, and 2.3-fold.

### 2.8. mRNA Levels of Bax and Bcl-2

**Figure 13:**
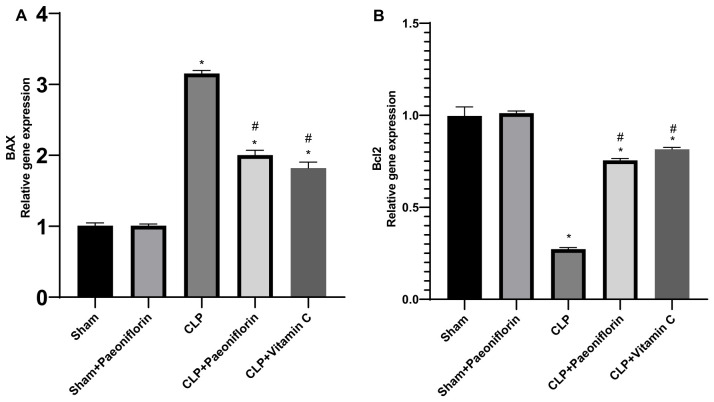
Effect of paeoniflorin on apoptosis-related gene expression. Relative mRNA expression levels of (**A**) Bax and (**B**) Bcl-2 in lung tissues. Data are expressed as fold change relative to the sham group and normalized to GAPDH (mean ± SD, *n* = 6). * *p* < 0.05 vs. sham group, # *p* < 0.05 vs. CLP group.

To examine whether paeoniflorin treatment reduced CLP induced apoptosis, Bax and Bcl-2 gene expressions were analyzed. As shown in Figure 13A, sepsis dramatically (*p* < 0.05) raised the Bax mRNA level to 3.15-fold relative to the sham group. This amplified mRNA level was dramatically (*p* < 0.05) attenuated (1.9-fold) in septic mice treated with paeoniflorin. Furthermore, the Bcl-2 mRNA level was significantly reduced compared with the sham mice (0.27-fold). However, pretreatment with paeoniflorin augmented this suppressed bcl2 (0.75-fold) in the lung tissues relative to the CLP group (*p* < 0.05), as illustrated in Figure 13B.

### 2.9. Protein Expression of the JAK2/STAT-3 Pathway

**Figure 14:**
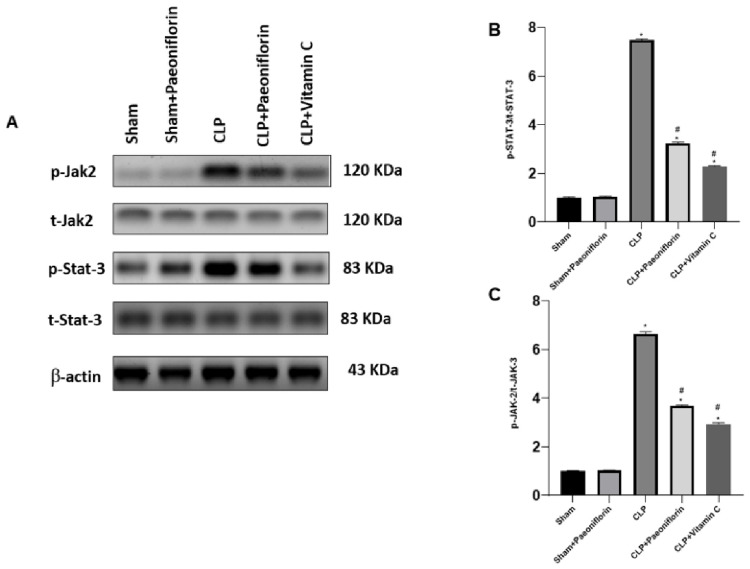
Effect of paeoniflorin on JAK2/STAT3 signaling pathway. (**A**) Representative Western blot bands of JAK2, p-JAK2, STAT3, p-STAT3, and β-actin. Quantitative analysis of (**B**) p-JAK2/t-JAK2 and (**C**) p-STAT3/t-STAT3 protein expression levels in lung tissue. Data are expressed as the mean ± SD (*n* = 6). * *p* < 0.05 vs. sham group, # *p* < 0.05 vs. CLP group.

The effect of paeoniflorin on the JAK2-STAT3 pathway in CLP mice was investigated using Western blot analysis. As displayed in Figure 14A,B, p-JAK-2/t-JAK-2 was dramatically greater in the septic animals (6.52-fold) relative to the sham-operated animals (*p*  <  0.05). On the contrary, paeoniflorin-administrated animals showed a suppressed CLP-induced increase in p-JAK-2/t-JAK-2 (3.71-fold) (*p*  <  0.05). Additionally, p-STAT3/t-STAT3 exhibited undeniable increases in the CLP group (7.45) relative to the sham animals (*p*  <  0.05), while paeoniflorin alleviated the CLP-induced increase in p-STAT3/t-STAT3 (*p*  <  0.05).

## 3. Discussion

The occurrence of sepsis is quite common and shows a steady increase, while the exact mechanisms behind its development remain a subject of ongoing research. Sepsis leads to damage in multiple organs, and ALI often occurs because of sepsis. The management of sepsis is inadequate; also, the mortality rate remains elevated [21].

Nevertheless, there is still much to learn about the way paeoniflorin counteracts lung injury. In the current study, we examined the effects of paeoniflorin on sepsis-induced ALI and delved into the mechanisms behind its actions. The integration of network pharmacology, molecular docking, and in vivo experiments provides a coherent mechanistic framework for interpreting our findings. Specifically, network pharmacology analysis served as a hypothesis-generating approach, identifying IL6, TNF, IL1B, and STAT3 as central hub genes potentially involved in the therapeutic effects of paeoniflorin in ALI. These targets guided the selection of key inflammatory mediators and signaling pathways for experimental validation. Consistent with these predictions, our in vivo results demonstrated significant downregulation of pro-inflammatory cytokines and inhibition of JAK2/STAT3 activation. Moreover, molecular docking analyses revealed favorable binding affinities of paeoniflorin toward the HIF-1, JUN, and inflammatory mediators, supporting the plausibility of direct molecular interactions. Collectively, these findings establish a mechanistic link between computational predictions and biological validation.

The determination of lung tissue capillary permeability was accomplished through the analysis of protein concentration in the BALF. In addition to total and differential cell counts, an assessment of the extent of lung inflammation was conducted. By quantifying the W/D ratio, the extent of edema in the lung tissue was also determined. Increases in the protein content, lung W/D weight ratio, total and differential cell counts in BALF were identified in the current experimental investigation of sepsis. It is noteworthy that paeoniflorin demonstrated the ability to mitigate the extent of inflammation in the lungs and alveoli through a reduction in the lung W/D weight ratio, total protein content, total and differential cell counts, and neutrophils. In addition, LDH activity was estimated in order to detect interstitial lung injury [22]. Our results demonstrated that sepsis increased LDH activity, indicative of greater interstitial lung injury, whereas treatment with paeoniflorin significantly decreased LDH activity. The results indicate that the administration of paeoniflorin significantly decreases vascular permeability, inflammation, and edema, while also enhancing the state of lung tissue. This implies that paeoniflorin may possess the capability to mitigate the pulmonary damage induced by sepsis.

The involvement of oxidants in inflammation and tissue injury is significant. Excessive oxidant production during sepsis disrupts the equilibrium between endogenous scavenging antioxidants and oxidants, thereby inducing oxidative stress [23]. Oxidative stress is a significant factor in the mortality rates associated with a wide range of diseases [23]. Sepsis is marked by an elevation in ROS production, which plays an essential part in the defense against bacterial infections. Nonetheless, increased levels of ROS significantly play a role in lung injury by triggering inflammatory responses within the lungs, leading to structural damage, injury to the alveoli, and heightened vascular permeability. The interplay of these factors plays a significant role in the inflammatory cascade and subsequent tissue damage [24]. During sepsis, antioxidant enzymes decrease, and lipid peroxidation increases, according to previous research [25,26]. Antioxidants therefore exert a significant influence on the progression of sepsis [25]. SOD, an enzymatic antioxidant, is particularly essential for granulocyte phagocytosis and the intracellular destruction of bacteria. As an important non-enzymatic antioxidant, GSH safeguards tissues against oxidative stress and maintains the integrity of proteins and lipids. MDA, an important consequence of lipid peroxidation, functions as a quantifier of ROS produced during lipid oxidation [27]. According to prior research, paeoniflorin can augment the antioxidant defense machinery by influencing antioxidant/oxidant parameters [28]. The current investigation revealed that the sepsis group exhibited a considerably greater suppression in SOD activity and GSH levels in the lung tissues relative to the sham group. Furthermore, the sepsis group displayed a significantly elevated concentration of MDA, thereby establishing the involvement of oxidative mechanisms in the tissue damage induced by sepsis. Administration of paeoniflorin led to a decrease in MDA levels within the pulmonary tissues of septic mice, while concurrently reinstating the GSH and SOD levels. This can prevent severe inflammation not only due to the antioxidant properties of paeoniflorin, but also by inhibiting the cytokine cascade, which is capable of causing severe damage. Previous research has demonstrated that lung injury induced by CLP was associated with a reduction in SOD activity and GSH levels, while the MDA levels increased [29,30]. The findings of this study are consistent with those earlier results. The findings were further corroborated by histopathological analyses.

The onset of ALI/ARDS entails an amplified and dysregulated inflammatory response of alveolar epithelial and capillary endothelial cells, instigated by diverse infectious or non-infectious agents [31]. ROS function as secondary messengers in intracellular signaling cascades. An excess of ROS triggers redox-sensitive transcription factors such as NF-κB [32]. These factors subsequently induce the synthesis of pro-inflammatory cytokines such as TNF-α, IL-1β, and IL-6, which in turn facilitate the injury and death of endothelial and epithelial cells in ALI pathogenesis, resulting in impairment of the alveolar-capillary barrier [31]. It has been observed that pulmonary vascular endothelial cells have the ability to suppress inflammation, attract immune cells, and control the migration of leukocytes in areas of inflammation [33]. When the endothelial barrier is compromised, chemokines are released, leading to the recruitment of neutrophils and macrophages. This intensifies a cycle of inflammation, resulting in heightened permeability of the endothelial cells due to the overproduction of oxygen free radicals [34]. To investigate the potential correlation between these cytokines and the protective properties of paeoniflorin, an examination was conducted on the levels of TNF-α, IL-6, and IL-1β in the lungs of mice. It was observed that paeoniflorin effectively inhibits the rise of these cytokines caused by sepsis. The data provide strong evidence that paeoniflorin has potential protective effects on septic lung injury in mice. It appears that these effects are achieved by inhibiting proinflammatory cytokines.

The advancement and evolution of lung damage in sepsis is intricately associated with cellular apoptosis in pulmonary tissues [35]. Studies have indicated that an overabundance of ROS can result in ALI, causing harm to cells, triggering pro-apoptotic signaling pathways, and ultimately resulting in the demise of alveolar epithelial and endothelial cells [36]. Bcl-2 and Bax play crucial roles in the process of apoptosis. Bcl-2 acts as a protein that prevents apoptosis, while Bax acts as a protein that promotes apoptosis [37,38]. The findings of this study indicate an increase in Bax and decrease in Bcl-2, resulting in the activation of caspase-3 and the subsequent initiation of apoptosis in the lungs. On the other hand, the procedure was reversed by paeoniflorin, indicating that paeoniflorin is associated with the modulation of apoptotic markers of pulmonary epithelial cells and provides protection against lung injury.

The JAK/STAT signaling pathways play a critical part in numerous biological as well as pathological processes, such as immune responses, maintenance of intracellular balance, cell growth, and cancer development [39]. The stimulation of the JAK/STAT pathway may enhance the expression of several associated cytokine receptors. These receptors can augment JAK activation upon binding to specific cytokines, leading to a “positive feedback” control of the JAK/STAT pathway. Prior research has indicated that various cytokines, such as TNF-α, IL-1β, and IL-6, can activate the JAK2/STAT3 signaling pathway [40,41]. Upon activation by various inflammatory stimuli, JAK-STAT facilitates the creation of receptor dimers and the phosphorylation of JAK subsequent to receptor contact on the cell membrane. The active JAK subsequently triggers the phosphorylation of STAT3 in the cytoplasm. The phosphorylated STAT3 molecules dimerize by binding through the SH2 structural domain. These dimers then translocate to the nucleus to regulate the expression of target genes [42,43]. The stimulation of the JAK/STAT pathway significantly amplifies the inflammatory response, serving a vital function in the initiation and advancement of sepsis [5]. Thus, it is crucial to consider interventions of JAK/STAT as potential targets for sepsis prevention and treatment. In the study, it was found that the phosphorylation of JAK2 and STAT3 proteins was minimal and their expression levels were low in the sham group. Following sepsis, the JAK/STAT signaling pathway in the lung becomes activated, as evidenced by the heightened levels of phosphorylation in JAK2 and STAT3. This finding aligns with the research performed by Severgnini et al., which suggests a potential link between STAT and the onset of ALI [44]. In a study conducted by Han et al., it was shown that the levels of JAK2/STAT3 were increased in a mouse model of severe acute pancreatitis-induced ALI [45]. Paeoniflorin, in contrast, effectively reduced the phosphorylation levels of JAK2 and STAT3, indicating that it may partially exert its protective efficacy by suppressing the JAK2/STAT3 pathway. While the present findings provide mechanistic insight into the protective effects of paeoniflorin, further experimental validation of additional predicted targets, such as HIF-1 and JUN, remains necessary to reinforce the network pharmacology outcomes and strengthen the integrative framework of the study. In addition, the use of targeted approaches, including selective pharmacological inhibitors or gene-silencing techniques, would be valuable to establish a definitive causal link for the involvement of the JAK2/STAT3 signaling pathway.

Another limitation of the current work is the evaluation of a single dosing regimen of paeoniflorin without a comprehensive dose–response assessment. Although the selected dose was guided by prior studies demonstrating efficacy in oxidative stress and inflammation-driven models, reliance on a single dose limits precise delineation of the exposure, response relationship, and the optimal therapeutic range [19,20]. Inclusion of multiple dose levels would allow for a more comprehensive characterization of the pharmacodynamic profile and enhance the translational significance of the findings. Furthermore, detailed pharmacokinetic studies are warranted to better characterize the absorption, distribution, and tissue exposure of paeoniflorin, particularly within pulmonary tissue. Accordingly, future investigations should integrate dose–response and pharmacokinetic analyses to provide a more robust foundation for its potential clinical application. Also, histopathological evaluation in this study was primarily descriptive and did not incorporate a standardized semi-quantitative scoring system or blinded assessment, which may limit objectivity. Future investigations should include validated lung injury scoring systems and blinded evaluation to enhance reproducibility and quantitative rigor.

## 4. Materials and Methods

### 4.1. Plant Material/Compound

Paeoniflorin (purity ≥ 98%), a major bioactive monoterpene glycoside isolated from the roots of *Paeonia officinalis* (family Paeoniaceae), was obtained commercially from Sigma–Aldrich (St. Louis, MO, USA). The compound was stored according to the manufacturer’s instructions and freshly prepared in the appropriate solvent prior to the experiment.

### 4.2. Computational Study

#### Network Pharmacology-Based Analysis

Genomic screening for targets associated to paeoniflorin

The target genes of compounds identified from paeoniflorin were derived by analyzing chemical similarities, pharmacophore models, and protein interactions, utilizing the Traditional Chinese Medicine Systems Pharmacology Database and Analysis Platform (TCMSP) database https://tcmsp-e.com/tcmsp.php (accessed on 25 May 2025) [46], the BATMAN-TCM platform (http://bionet.ncpsb.org.cn/batman-tcm/ (accessed on 25 May 2025)) [47], and the SwissTargetPrediction database (http://www.swisstargetprediction.ch/ (accessed on 25 May 2025)). These databases were selected due to their complementary approaches, including experimentally validated interactions and predictive modeling based on chemical similarity.

Targets were collected based on default database criteria, and duplicate entries were removed to ensure data consistency. Following this step, the identified target genes were transformed into their corresponding canonical gene names with the assistance of the UniProt database (https://www.uniprot.org/ (accessed on 25 May 2025)) [48].

Screening of acute lung injury related target genes

Genes linked to ALI were sourced from the National Center for Biotechnology Information (NCBI) (https://www.ncbi.nlm.nih.gov (accessed on 25 May 2025)), GeneCards (https://www.genecards.org/ (accessed on 25 May 2025)) [49], and DisGeNET database (https://www.disgenet.org/ (accessed on 25 May 2025)) using the keywords “acute lung injury” and restricting the search to the species “*Homo sapiens*”. To improve specificity, genes with higher relevance scores (where applicable, such as in GeneCards) were prioritized. Redundant targets were eliminated, and overlapping component-related and disease-related proteins were recognized through the utilization of InteractiVenn (https://www.interactivenn.net/ (accessed on 25 May 2025)) [50] intersections as potential targets of these components in ALI.

Construction of protein–protein interaction (PPI) network

A PPI network was constructed using STRING version 12.0 (https://string-db.org/) [51] with the organism set to *Homo sapiens* and a minimum required interaction score of 0.400 (medium confidence) based on a query of target genes. The cutoff value of 0.400 was selected as it represents a medium confidence level in STRING, allowing for the inclusion of biologically meaningful interactions while avoiding excessive low-confidence noise. The network was then exported to Cytoscape 3.10.1 (Seattle, WA, USA) [52], an open-source platform for viewing, modeling, and studying molecular and genetic interaction networks. The top ten important genes were selected using the CytoHubba plug-in based on degree centrality analysis, which ranks nodes according to their number of interactions within the network.

Molecular docking

The crystal structures of seven potential target genes were retrieved from the Protein Data Bank (PDB) including IL6 (pdb: 1ALU), IL-1β (pdb: 6Y8M), TNF-α (pdb: 2AZ5), HIF-1 (pdb: 3KCX), STAT3 (pdb: 6NJS), JUN (pdb: 2P33), and Bax (pdb: 1F16) [53]. These targets were selected based on their identification as hub genes in the PPI network and their relevance to inflammatory and JAK2/STAT3 signaling pathways. Input files for the discovered chemical compounds, protein structures, and co-crystallized ligands were generated using AutoDock Tools (version 1.5.7) [54]. Additionally, all ligands were processed using OpenBabel, version 3.1.1 [55]. Molecular docking of the chemical compounds to the target proteins was performed using AutoDock Vina [56]. Grid boxes were defined to encompass the active site of each target protein, with volumes not exceeding 27,000 Å^3^, and the exhaustiveness parameter was set to 8. To validate the docking procedure, co-crystallized ligands were re-docked into the crystal structures of proteins bound to their ligands, and the root-mean-square deviation (RMSD) between the docked and crystallographic poses was confirmed to be less than 2 Å, indicating acceptable accuracy of the docking protocol. The interactions of the docked compounds with important proteins were studied and assessed using Discovery Studio Visualizer 21.1.0.2.

### 4.3. Animals

Adult male Wistar albino mice, (6 to 9 weeks of age), were obtained from the experimental animal laboratory of the Deraya Center for Scientific Research at Deraya University. One week prior to the experiment, the rodents were provided unrestricted access to food pellets and water to facilitate their acclimatization to the laboratory milieu. The subjects were housed under controlled conditions featuring a 12-h light–dark cycle, a temperature set at 25 ± 2 °C, and a relative humidity of 45 ± 5%. Our study was approved by the Ethical Committee of the Deraya Center for Scientific Research (approval number: DCSR-01024-03).

### 4.4. Experimental Design

Thirty mice were randomly allocated into five groups, with each group consisting of six mice (*n* = 6): (1) the sham group; (2) the paeoniflorin group; (3) the group that underwent CLP (i.e., the CLP group); (4) the group treated with paeoniflorin and CLP; and (5) the group that received vitamin C and CLP, which was the standard group. Paeoniflorin, obtained from Sigma Aldrich (St. Louis, MO, USA), was administered orally at a dosage of 40 mg/kg per day for 4 d. On day 4, the CLP procedure was conducted. The mice were given a single intraperitoneal dosage of vitamin C (200 mg/kg) 4 d before the CLP operation [57]. In the sham group, mice were administered 200 μL of normal saline orally on a daily basis for 4 d. On the 4th day, a CLP operation was performed without actually carrying out the CLP procedure. The CLP group received an equivalent amount of normal saline (200 μL) orally. The mice were administered thiopental (50 mg/kg) to induce anesthesia and were euthanized 24 h after the procedure. Then, lung tissue and bronchoalveolar lavage fluid (BALF) were collected. One lobe of the lung was kept in formalin for later histological examination. The second lobe of the lung was subjected to preservation at −80 °C for the purpose of conducting additional biochemical studies. The third lobe, on the other hand, was utilized to determine the ratio of wet to dry (W/D) weight.

### 4.5. CLP Model and Tissue Collection

The CLP technique, as noted earlier, was utilized to induce sepsis [58,59]. The CLP technique was employed to induce polymicrobial sepsis of moderate-to-severe grade, as previously described with modifications. Briefly, animals were anesthetized with an intramuscular injection of ketamine (100 mg/kg) and xylazine (10 mg/kg). After abdominal shaving, disinfection with 70% ethanol, and midline laparotomy, the cecum was carefully exteriorized. Ligation was performed at 75% of the total cecal length distal to the ileocecal valve using a 3-0 non-absorbable suture (0.3 mm diameter), preserving intestinal continuity and mesenteric blood supply. Two full-thickness punctures were then made through the antimesenteric wall of the ligated cecum using an 18-gauge needle, and a small amount of fecal material was expressed through each puncture site to confirm patency. The cecum was returned to the abdominal cavity, and the abdominal wall was closed in two layers. All CLP mice received subcutaneous saline (1 mL/100 g) immediately post-operatively for fluid resuscitation. Sham-operated mice underwent identical procedures, including laparotomy and cecal exteriorization, without ligation or puncture. Animals were euthanized 24 h post-procedure under thiopental anesthesia (50 mg/kg, i.p.), and lung tissue and BALF were immediately collected. The 24-h endpoint was selected based on established evidence of peak acute-phase inflammatory and oxidative injury in this model [60].

### 4.6. Survival Analysis

A further group of 50 mice was assembled and subjected to identical treatment protocols (*n* = 10). We undertook systematic daily observations and meticulously documented the count of deceased mice within each group over a span of 7 d. The mortality rate was ascertained throughout the duration of the observation period.

### 4.7. Histological Analysis

The lung tissues were immersed in paraformaldehyde for 24 h to ensure proper fixation. Following the dehydration process, the specimens were embedded in paraffin and dissected into 5-μm sections. The subsequent observation of pathological changes was carried out using hematoxylin–eosin (H&E) staining.

### 4.8. BALF (Bronchoalveolar Lavage Fluid) Analysis

BALF was collected by tracheal cannulation and three successive instillations of ice-cold sterile PBS (0.5 mL each; total 1.5 mL per animal), with a recovery rate of consistently ≥ 85%. The pooled BALF was centrifuged at 400× *g* for 10 min at 4 °C. The cellular pellet was resuspended in 500 μL sterile PBS, placed onto slides by cytospin, and stained with Wright-Giemsa for 8 min. Total and differential cell counts were performed using a Neubauer hemocytometer and expressed as cells/mm^3^ (×10^3^). A minimum of 200 cells per slide were counted by two blinded independent observers, with the proportion of neutrophils calculated as a percentage of total cells. The acellular supernatant was stored at −80 °C for protein and LDH analyses. Total protein concentration was measured by the Bradford assay (Bio-Rad, Hercules, CA, USA) using a BSA standard curve (0.2–1.4 mg/mL; R^2^ ≥ 0.99) with absorbance at 595 nm. LDH activity was measured using a colorimetric kit (Biodiagnostic, Giza, Egypt; Cat. No.: LD 25 19), based on NAD^+^ reduction at 340 nm, with a linear detection range of 10–500 IU/L and a detection limit of 5 IU/L. Results are expressed as IU/L.

### 4.9. The Proportion of W/D Weight

The calculation of extravascular lung water functions as a marker for lung edema. One lobe of the lung was removed, and its mass was measured. The lung was subsequently placed in an incubator at 80 °C for 24 h to determine the dry weight. The W/D value was determined by the quotient of wet weight and dry weight.

### 4.10. Oxidative Stress Assessment

Lung tissue was homogenized in ice-cold PBS (10% *w*/*v*, pH 7.4) at 10,000 rpm for 30 s and centrifuged at 3000× *g* for 15 min at 4 °C. Supernatants were used for all oxidative stress assays, with results normalized to total protein content (Bradford assay). Pulmonary MDA levels were quantified by the TBARS assay (Biodiagnostic, Cat. No.: MD 25 29) at 534 nm using a 1,1,3,3-tetraethoxypropane standard curve (0.5–10 nmol/mL; LOD: 0.1 nmol/mL; R^2^ ≥ 0.99); results expressed as nmol/mg protein. GSH was measured by DTNB colorimetric assay (Biodiagnostic, Cat. No.: GR 25 11) at 405 nm using a GSH standard curve (0.1–2.0 mmol/L; LOD: 0.05 mmol/L; R^2^ ≥ 0.99); results expressed as mmol/mg protein. SOD activity was determined by the pyrogallol inhibition assay (Biodiagnostic, Cat. No.: SD 25 21) at 480 nm; one unit was defined as 50% inhibition of auto-oxidation under standard conditions and the results expressed as U/mg protein.

### 4.11. Gene Expression Analysis

RNA was isolated from lung tissues with the aid of Trizol reagent (Thermo Fisher Scientific, Waltham, MA, USA) [61]. The RNA purity was assessed by a NanoDrop^®^ spectrophotometer (Thermo Fisher Scientific, Waltham, MA, USA). The cDNA was synthesized employing the RevertAid^®^ First Strand cDNA Synthesis Kit from Thermo Fisher Scientific. A real-time PCR experiment was conducted utilizing the StepOne system from Thermo Fisher Scientific. The Maxima SYBR Green Master Mix from Thermo Fisher Scientific was used.. Quantification was performed using the comparative CT method, with normalization to GAPDH and calculation as follows: RQ = 2^−ΔΔct^ [62].

### 4.12. Western Blotting

The proteins in lung tissues were resolved using SDS-polyacrylamide gel electrophoresis (PAGE) in 10% polyacrylamide gels then transferred onto a PVDF membrane. Following the membrane blocking step, the presence of the relevant protein was assessed with specific antibodies. The antibodies utilized in this study were JAK2, ph-JAK2, STAT3, ph-STAT3, and β-actin. The goat anti-rabbit IgG antibody was obtained from Cell Signaling Technology (Boston, MA, USA). An imaging technique involving a CCD camera was utilized, and the produced images were evaluated by ImageJ 1.46r software (National Institutes of Health, Bethesda, MD, USA). [63].

### 4.13. Statistical Analysis

Data are presented as means ± standard deviation (SD) and were analyzed using GraphPad Prism software ((Version 9.5.1, GraphPad Software, San Diego, CA, USA). Prior to analysis, data distribution was assessed for normality using the Shapiro–Wilk test, and the homogeneity of variances was evaluated using Levene’s test. For normally distributed data with equal variances, group comparisons were performed using one-way analysis of variance (ANOVA) followed by Tukey’s multiple comparisons test. In cases where the data did not meet the parametric assumptions, appropriate non-parametric tests (Kruskal–Wallis test followed by Dunn’s post hoc test) were applied. Multiple comparisons were controlled using Tukey’s or Dunn’s post hoc corrections as appropriate. A *p*-value < 0.05 was considered statistically significant.

## 5. Conclusions

In summary, the findings of this study indicate that administering paeoniflorin significantly reduces acute lung injury caused by CLP and improves the survival rates in mice. Notably, this investigation is the first to reveal that paeoniflorin exerts a protective effect on lung tissue in a sepsis model induced by CLP, primarily by addressing oxidative stress, inflammation, and modulating the JAK-STAT signaling pathway. Hence, paeoniflorin shows potential as a therapeutic option for addressing oxidative stress and the hyperinflammatory phase of sepsis. Network pharmacology analysis was employed to elucidate the mechanisms and pathways underlying the protective role of paeoniflorin in acute lung injury. Molecular docking simulations indicated that paeoniflorin exhibits strong affinity for HIF-1 and JUN, as well as moderate affinities for IL-1β, TNF-α, and Bax, highlighting its potential therapeutic applications in modulating hypoxia responses, gene expression, and inflammatory and apoptotic pathways. Future research should aim to further elucidate the therapeutic potential of paeoniflorin and explore its clinical applicability. Investigating the specific interactions between paeoniflorin and key components of the JAK2/STAT3 pathway, such as the phosphorylation dynamics and downstream transcriptional targets, will provide a more comprehensive understanding. Detailed pharmacokinetic studies are essential to determine the absorption, distribution, metabolism, and excretion (ADME) profile of paeoniflorin. This will help optimize dosing regimens and assess its bioavailability.

## Figures and Tables

**Table 1:** Topological parameters of the top ten hub genes.

No.	Name	Target	Degree	Betweenness	Closeness
1	Interleukin-6	IL6	16	0.144	1
2	Tumor necrosis factor	TNF	15	0.078	0.941
3	Interleukin-1 beta	IL1B	14	0.088	0.889
4	Hypoxia-inducible factor 1-alpha	HIF1A	13	0.033	0.842
5	Signal transducer and activator of transcription 3	STAT3	13	0.024	0.842
6	Nuclear factor NF-kappa-B p105 subunit	NFKB1	13	0.024	0.842
7	C-C motif chemokine 2	CCL2	12	0.019	0.800
8	Cytochrome b-245 heavy chain	CYBB	11	0.007	0.762
9	Interleukin-8	CXCL8	11	0.014	0.762
10	NADPH oxidase 4	NOX4	9	0.001	0.696

**Table 2:** Docking scores of the tested compounds.

Compound	Docking Score (kcal/mol)						
	IL6 (1ALU)	IL-1β (6Y8M)	TNF-α (2AZ5)	HIF-1 (3KCX)	STAT3 (6NJS)	JUN (2P33)	Bax (1F16)
Paeoniflorin	−2.20	−4.03	−4.36	−5.25	−3.69	−5.80	−4.38
Co-Crystallized Ligand	−5.14 RMSD = 1.46 Å	−5.62 RMSD = 0.193 Å	−5.173 RMSD = 1.40 Å	−5.11 RMSD = 0.58 Å	−10.01 RMSD = 1.96 Å	−10.21 RMSD = 1.40 Å	------

**Table 3:** Summary of molecular docking interactions between paeoniflorin and target proteins.

Label	Target	Hydrogen Bonds (H-Bonds)	Other Interactions
A	IL-1β	ASN108, GLN149, THR147, GLN15, MET148 and LYS109	LEU110 (hydrophobic) and MET148 (hydrophobic)
B	TNF-α	GLN61, TYR119 and GLU146	TYR119 (hydrophobic/π interaction)
C	HIF-1	ASP201, GLN147 and PHE100	TYR102 (π–π stacking), TRP296 (hydrophobic interaction), HIS199 (hydrophobic interaction), LEU188 (hydrophobic interaction)
D	JUN	ASP150 and ASN152	VAL 78, LEU 206, and LYS 93 (hydrophobic interaction), MET 146 (sulfur–π interaction)
E	Bax	VAL83, GLN77, LYS 119 and LYS123	ALA81, ILE80 and LEU120 (hydrophobic)

## Data Availability

The original contributions presented in this study are included in the article. Further inquiries can be directed to the corresponding author.
